# Effects of toner-handling work on respiratory function, chest X-ray findings, and biomarkers of inflammation, allergy, and oxidative stress: a 10-year prospective Japanese cohort study

**DOI:** 10.1186/s12890-020-01320-6

**Published:** 2020-10-27

**Authors:** Niina Terunuma, Kazunori Ikegami, Hiroko Kitamura, Hajime Ando, Shizuka Kurosaki, Masashi Masuda, Takeshi Kochi, Nobuaki Yanagi, Yoshihisa Fujino, Akira Ogami, Toshiaki Higashi

**Affiliations:** 1 Department of Work Systems and Health, Institute of Industrial Ecological Sciences, University of Occupational and Environmental Health, Kitakyushu, 807-8555 Japan; 2 Human Resources Department, AEON Co. Ltd., Chiba, 261-8515 Japan; 3 Department of Environmental Epidemiology, Institute of Industrial Ecological Sciences, University of Occupational and Environmental Health, Kitakyushu, 807-8555 Japan

**Keywords:** Cohort study, Laser printer, Occupational health, Photocopier, Pneumoconiosis, Toner, Toner-handling work, Biomarkers, Respiratory function

## Abstract

**Background:**

Exposure to toner, a substance used in photocopiers and printers, has been associated with siderosilicosis and other adverse effects. However, these findings are limited, and there is insufficient evidence on the long-term effects of toner exposure. Using longitudinal analysis, this study aimed to examine the effects of work involving toner exposure on the respiratory system over time.

**Methods:**

We conducted a prospective cohort study in a Japanese toner and copier manufacturing enterprise between 2003 and 2013. The cohort included a total of 1468 workers, which comprised 887 toner-handling workers and 581 non-toner-handling workers. We subdivided the toner-handling workers into two groups according to the toner exposure concentration, based on the baseline survey in 2003. We compared the chest X-ray results, respiratory function indicators, and serum and urinary biomarkers of inflammation, allergy, and oxidative stress among the three groups: high-concentration toner exposure group, low-concentration toner exposure group, and non-toner-handling group. To consider the effects of individual differences on the longitudinal data, we used a linear mixed model.

**Results:**

Similar chest X-ray results, the biomarkers, and most of the respiratory function indicators were found in the non-toner-handling and toner-handling groups. There were no significant yearly changes in the percentage of vital capacity (%VC) in the high-concentration toner exposure group, while there was a significant yearly increase in %VC in the low-concentration toner exposure group and non-toner-handling group. The yearly change in each group was as follows: high-concentration toner exposure group, − 0.11% (95% confidence interval [CI], − 0.29 to 0.08; *P* = 0.250); low-concentration toner exposure group, 0.13% (95% CI, 0.09–0.17; *P* < 0.001); and non-toner-handling group, 0.15% (95% CI, 0.01–0.20; P < 0.001).

**Conclusions:**

In our 10-year prospective study, toner-handling work was not associated with the deterioration of respiratory function and an increase in biomarker values for inflammation, allergy, and oxidative stress. This finding suggests that toner-handling work is irrelevant to the onset of respiratory disease and has minimal adverse effects on the respiratory system under a well-managed work environment.

**Supplementary Information:**

**Supplementary information** accompanies this paper at10.1186/s12890-020-01320-6.

## Background

Toner, a particulate substance, with a diameter of 5–10 μm, is used in photocopiers and laser printers to form a printed image or text on paper. The inside of a toner resin particle contains colorants such as carbon black, whereas the surface of the particle contains nanoparticle additives such as titanium dioxide and amorphous silica. In 1994, Gallardo et al. reported the first case of siderosilicosis owing to toner exposure, and since then, there have been further case reports of sarcoidosis, allergic rhinitis, asthma, etc., being associated with toner exposure [1–4]. As the use of photocopiers, printers, and toners has increased, their respiratory effects have been highlighted. Recent studies have shown that office machines such as printers or photocopiers can emit particulate matter (PM) when in use, and PM may cause indoor air pollution [5–7]. However, studies on emissions from laser printers suggested that these emitted particles have different characteristics from toner dust itself, such as particle size at the sub-micron level, volatility, and being composed of semi-volatile organic compounds [5–11]. The degree of toxicity of PM is related to the physicochemical properties of the particles and their particle size. Thus, it is necessary to assess the health effects of toner exposure and those of PM emitted from office machines separately.

Several previous studies have reported the health effects of toner exposure in toner-manufacturing workers and suggest that toner particle inhalation has potential adverse effects [12–15]. However, these studies were limited owing to the statistical analysis methods, sample sizes, and other factors. Moreover, there is insufficient information on the long-term health effects of toner exposure.

We commenced a 10-year cohort study regarding the respiratory health effects of working in Japanese toner and copier manufacturing enterprise in 2003. In the results of this cohort study, the effects of toner-handling work on the incidence of lung diseases and changes in the prevalence of subjective respiratory symptoms have already been published [16]. The purpose of this paper is to report the effects of toner-handling work on the findings of chest X-ray, respiratory function tests, and serum or urinary biomarker tests using longitudinal analysis.

## Methods

### Study design and setting

This prospective cohort study was conducted across successive 10 years. We conducted a baseline survey in 2003 and implemented follow-up surveys yearly from 2004 (first survey) to 2013 (tenth survey). Each participant received a periodic health check and completed 1) a toner-handling work status survey, 2) a questionnaire-based survey on self-reported respiratory symptoms and diseases, 3) chest radiography, 4) respiratory function tests, and 5) serum and urinary biomarker tests. We particularly examined the effects of toner-handling work on chest X-ray findings, respiratory function, inflammation, allergy, and oxidative stress.

### Sample size calculation

The incidence of respiratory disease associated with toner exposure is not well known. Therefore, assuming that the prevalence of abnormal chest X-ray findings among the background characteristics was about 50 out of 100,000 toner-handling workers, and that the prevalence when the effect of toner exposure is significant is about 150 out of 100,000 toner-handling workers, we would need about 2100 toner-handling-workers based on 90% power and 5% level of significance. When the background prevalence was set at < 10 out of 100,000 toner-handling workers, about 860 toner-handling workers were needed. We therefore estimated that it would be desirable to have about 1000 toner-handling-workers in this study.

### Participants

A total of 918 participants who were 19–50 years of age in 2003, worked in one toner and copier manufacturing enterprise, and handled toner particles at work, were potentially eligible for this study (toner-handling group). Their toner-handling work included toner development, toner manufacturing, toner or copy machine development, toner or copy machine recycling, and customer service. Additionally, we recruited gender-matched non-toner-handling workers aged 19–50 years who also worked in the same business sites as those in the toner-handling group. A total of 586 non-toner handlers were enrolled as controls (non-toner-handling group). We confirmed that the control group mainly engaged in desk work not often involving copy printing and had never engaged in toner-handling work. The toner-handling area and the area where the control group worked were physically separated. Participants were excluded from the analysis if they lacked a detailed work history or if they had already been diagnosed with chronic granulomatous pneumonia, pneumoconiosis, or lung cancer at the time of the baseline survey.

### Chest X-ray examination

We performed a yearly chest X-ray examination on each participant following the standard examination method regulated by the Pneumoconiosis Law in Japan [17, 18]. The chest X-ray images were interpreted following the panel reading by two skilled readers, based on the international classification of pneumoconiosis (a 12-point scale from 0/− to 3/+) [19], and were electronically stored using a film digitizer. To avoid differential misclassification, the readers of the X-ray images were not given information about the toner-handling status of the participants.

### Respiratory function tests: spirometry and flow-volume curve

We conducted yearly respiratory function tests for each participant, including the following parameters: vital capacity (VC), percentage of VC to predicted VC value (%VC), forced expiratory volume in 1 s (FEV_1_), percentage of FEV_1_ to predicted FEV_1_ value (%FEV_1_), percentage of forced expiratory volume in 1 s to forced vital capacity (FEV_1_/FVC), percentage of FEV_1_/FVC to predicted FEV_1_/FVC value (%FEV_1_/FVC), maximal expiratory flow at 25% FVC (V25), and percentage of V25 to predicted V25 value (%V25). The respiratory function tests were performed using Microspiro HI-701 and Microspiro HI-801 (CHEST Corporation, Tokyo, Japan), which are pneumotach-type spirometry measuring units that meet the standards regulated by the American Thoracic Society [20]. We measured each parameter three times on the same day to obtain adequate values. To ensure consistent and valid measurement, a skilled examiner at the same medical institution conducted the respiratory function tests throughout each 1-yearly study period. We calculated the predicted values for VC, FEV_1_, FEV_1_/FVC, and V25 for each participant using the formula based on sex, age, and height indicated by the Japanese Respiratory Society [21, 22].

### Serum and urinary biomarker tests

Each participant underwent yearly biomarker tests for inflammation, allergy, and oxidative stress, such as those for C-reactive protein (CRP), immunoglobulin E (Ig E), interleukin (IL)-4, IL-6, IL-8, and interferon-gamma (IFN-γ) in serum, and 8-hydroxy-2′-deoxyguanosine (8-OHdG) in urine. To maintain accuracy and precision throughout the whole survey, we requested the OHG Institute Co., Ltd. (Kitakyushu, Japan), to perform the analysis of 8-OHdG, and SRL Inc. (Tokyo, Japan) to analyze other biomarkers.

We used latex immunoagglutination assays for analyzing CRP; fluorescent enzyme immunoassays for IgE; chemiluminescent enzyme immunoassays for IL-4 and IL-6; enzyme-linked immunosorbent assays for IL-8; enzyme immunoassays for IFN-γ; and high-performance liquid chromatography for 8-OHdG. Spot urinary 8-OHdG concentrations could be unstable due to the participants’ physical activity intensity, urine collection time, and other factors. Hence, creatinine-corrected 8-OHdG values were adopted in this study. The limits of detection (LODs) at SRL Inc. were 0.02 mg/dL for CRP, 5.00 IU/mL for IgE, 2.00 pg/mL for IL-4, 0.20 pg/mL for IL-6, 2.00 pg/mL for IL-8, and 0.10 IU/mL for IFN-γ. We allotted the values of LOD/2 to the undetectable values of each biomarker.

### Toner particle

The toner-handling workers were exposed to two types of toner particles during the study period. Convention toner (C toner) and emulsion aggregation toner (EA toner) were manufactured (C toner is produced by pulverizing raw materials) in the toner- and copy- machine-manufacturing enterprise wherein this study was conducted. This factory produced less EA toner than C toner from 2004 to 2006. However, the proportion of production was reversed in 2007; the production of EA toner steadily increased [23].

The mean particle diameters of the C and EA toners manufactured by this enterprise were 6.5 μm and 5.8 μm, respectively. Black C toner is composed of 70–80% polyester resin, 10–20% ferrite powder (iron oxide and manganese oxide), < 10% amorphous silica, < 10% carbon black, and < 1% titanium dioxide. Black EA toner is composed of 60–70% styrene-acrylate resin, 10–20% ferrite powder (iron oxide and manganese oxide), < 10% polyethylene, < 10% amorphous silica, < 10% carbon black, and < 1% titanium dioxide [24].

### Toner exposure assessment

We have previously reported our findings following detailed assessments of toner exposure levels [19, 21–24]. In particular, Matsuda et al. described the details of the actual state of toner exposure in workers who handled toner in the same enterprise where this study was conducted.

In previous studies [23, 25–28], participants were randomly selected from among workers who engaged in five categories of work. Their toner exposures were measured using a personal dust sampler every year between 2003 and 2011. In fiscal years 2003 and 2004, we used a Roken-type Filter Holder for Personal Total and Respirable Dust Sampler (Model PS-43; Shibata Scientific Technology Ltd., Soka, Saitama, Japan) to measure the particles. These samplers were equipped with glass-fiber filters (PTFE binding and T60A20 type ϕ 25 mm; Tokyo Dylec Corp., Tokyo, Japan). An AirChek 2000 Sample Pump (SKC Inc., Pennsylvania, USA) or Gilian GilAir-5 Air Sampling Pumps (Sensidyne, St. Petersburg, Florida, USA) was used, with a flow rate of 1.5 L/min. These instruments collected particles with a size classification that was characteristically set at 5 μm (50% cutoff-point). In the fiscal years 2005 to 2010, we used a Model NWPS-254 Filter Holder for Personal Dust Sampler (Shibata Scientific Technology). This sampler was equipped with glass-fiber filters (PTFE binding and T60A20 type ϕ 25 mm; Tokyo Dylec.), and AirChek 2000 Sample Pumps or Gilian GilAir-5 Air Sampling Pumps were used, with a flow rate of 2.5 L/min. These instruments collected particles with a size classification that was characteristically set at 4 μm (50% cutoff- point).

The levels of personal exposure to toner particles were different for each type of toner-handling work; being significantly higher in machine-recycling work and toner-manufacturing work than in three other types. The mean 8-h time-weighted average (TWA-8 h) (SD) of each worker according to the five types of toner-handling work at the baseline survey was 0.989 (0.786) mg/m^3^ for toner and copy machine recycling (hereafter referred to as “recycling”), 0.203 (0.441) mg/m^3^ for toner manufacturing, 0.034 (0.030) mg/m^3^ for toner development, 0.019 (0.063) mg/m^3^ for toner and copy machine development, and 0.020 (0.060) mg/m^3^ for customer service. In all types of toner-handling work, the TWA-8 h value was much lower than the 3.0 mg/m^3^ maximum level allowed for unspecified particles, defined as the threshold limit value–time-weighted average (TLV-TWA), recommended by the American Conference of Governmental Industrial Hygienists (ACGIH) [29].

### Subgrouping according to toner exposure assessment

We divided the toner-handling group into two groups based on the toner exposure assessment, namely the high-concentration toner exposure group, who engaged in recycling and toner manufacturing, and the low-concentration toner exposure group, who engaged in the other three types of toner-handling work. Thus three groups in total were created, including the non-toner-handling group. We then evaluated the health effects of toner particle exposure among the three groups.

### Statistical analysis

To compare between two independent groups, qualitative variables were analyzed using the chi-square test or Fisher’s exact test, and quantitative variables were analyzed using the simple t-test and Welch’s t-test. The mean values of each parameter over the 10-year period were compared between the two groups by performing a two-way repeated measures analysis of variance with each parameter as the dependent variable and toner handling status as the independent variable. We used a linear mixed model (LMM) [30] to analyze the longitudinal change. Dependent variables consisted of the respiratory function test parameters and the biomarker values, and the following four models were analyzed. In model 1, we treated toner-handling work, the survey year, and the interaction between toner-handling work and survey year as fixed effects and treated only the individual differences at baseline as the random effects (random intercept model). In model 2, we added both individual differences at baseline and responses to toner exposure as random effects (random intercept and slope models). Akaike’s Information Criterion (AIC) was used to determine the model with high fitness. In model 3, we adapted a model with lower AIC values, and adjusted the model using age at baseline, body mass index, smoking, asthma, allergic rhinitis, pneumonia, sinusitis, exposure to dust other than toner at work, and organic solvent-handling work as confounding factors. Baseline surveys [25, 31] and interim reports [26–28] have suggested that these variables may influence the dependent variables. Additionally, in model 4, with regard to toner-handling work, analysis was performed using the three groups, that is, the high-concentration toner exposure group, low-concentration toner exposure group, and non-toner-handling group. We also adapted a higher-fit model of the random intercept model and the random intercept and slope model for model 4.

If any significant effects of toner exposure on each parameter were observed, we also performed LMM analysis adjusted for the same confounding factors as models 3 and 4, respectively for each exposure concentration level group. In all analyses, the threshold for significance was at *P* < 0.05. IBM SPSS Statistics for Windows 23(IBM Corp., Armonk, N.Y., USA) was used.

Definition of confounding factors.

Individuals who declared that they were currently smoking were considered as smokers. Those who had never smoked and those who had quit smoking before the study began were considered as non-smokers. The presence or absence of asthma, allergic rhinitis, pneumonia, and sinusitis, which were included as confounding factors in the statistical analyses, were self-reported by the participants. The medical history of pneumonia was investigated with the intention of community-acquired pneumonia and did not include chronic granulomatous pneumonia.

## Results

### Participants

Although gender was not an exclusion criterion; however since all the toner handling workers were males, the control group was also recruited from among the male workers. Therefore, all the participants were males. Among 1504 participants, 9 toner handlers and 2 non-toner handlers withdrew their participation from this study before the baseline survey. The reasons for the withdrawal were not related to the onset of respiratory disorder. The number of participants in the baseline survey was 909 for the toner-handling group and 584 for the non-toner-handling group. We excluded 25 participants (22 toner handlers and 3 non-toner handlers) who enrolled in the baseline survey from analysis, owing to the deficiency of work history data. Finally, we analyzed the data of 1468 participants (887 for the toner-handling group and 581 for the non-toner-handling group). None of them had a history of chronic granulomatous pneumonia, pneumoconiosis, or lung cancer at the baseline survey.

On average, the participants completed 8.8 out of 10 follow-up surveys. The average length of follow-up (from the baseline survey to the last follow-up survey) was 8.9 years. There were no significant differences in these parameters between the toner-handling group and non-toner-handling group. Baseline characteristics of the participants are shown in Table 1. Of the 887 participants in the toner-handling group, 49 participants, who worked in the recycling process and toner manufacturing process, were assigned to the high-concentration toner exposure group while the other 838 participants were assigned to the low-concentration toner exposure group. Table 2 shows the descriptive data for the high-concentration and the low-concentration toner exposure groups.

**Table 1:** Baseline characteristics of study participants

Parameters (unit)	Toner-handling group		Non-toner-handling group		*P*-value
	*n* = 887		*n* = 581		
	M (SD)/%		M (SD)/%		
Age (years)	38.7	(7.1)	38.5	(6.5)	0.678
BMI (kg/m^2^)	23.3	(2.9)	23.7	(2.9)	0.023
Current smoker	51.2%		46.8%		0.109
Prevalence of respiratory diseases (%)					
Asthma	9.5%		10.3%		0.591
Allergic rhinitis	51.1%		48.2%		0.286
Allergic dermatitis	20.9%		22.2%		0.558
Pneumonia	0.5%		0.3%		1.000
Sinusitis	2.7%		1.9%		0.383
Ratio of workers handling harmful substances (%)					
Workers handling dust other than toner	2.9%		0.3%		< 0.001
Workers handling organic solvent	26.3%		2.9%		< 0.001
Respiratory function test indicators					
VC (l)	4.2	(0.6)	4.2	(0.6)	0.390
%VC (%)	92.4	(10.7)	91.8	(9.8)	0.307
FEV_1_ (l)	3.5	(0.5)	3.5	(0.5)	0.521
%FEV_1_ (%)	95.0	(11.2)	94.5	(10.8)	0.412
FEV_1_/FVC (%)	83.1	(5.9)	82.9	(6.0)	0.655
%FEV_1_/FVC (%)	100.1	(6.9)	100.1	(7.1)	0.938
V25 (l/s)	1.6	(0.5)	1.6	(0.5)	0.940
%V25 (%)	68.2	(19.9)	67.4	(18.7)	0.413
Biomarkers					
CRP (mg/dl)	0.09	(0.18)	0.10	(0.21)	0.455
IgE (IU/ml)	201.6	(380.3)	219.9	(436.6)	0.424
IL-4 (pg/ml)	19.2	(29.4)	10.8	(31.1)	< 0.001
IL-6 (pg/ml)	1.40	(3.20)	1.20	(4.70)	0.412
IL-8 (pg/ml)	2.90	(19.90)	1.10	(0.80)	0.017
IFN-γ (IU/ml)	0.11	(0.01)	0.07	(0.07)	< 0.001
8-OHdG/Creatinine (ng/mg)	3.90	(1.50)	3.80	(1.4)	0.357

*M* mean, *SD* standard deviation, *BMI* body mass index, *VC* vital capacity, *%VC* percentage of VC to predicted VC value, *FEV*_*1*_ forced expiratory volume in 1 s, *%FEV*_*1*_ percentage of FEV_1_ to predicted FEV_1_ value, *FEV*_*1*_*/FVC* percentage of forced expiratory volume in 1 s, *%FEV*_*1*_*/FVC* percentage of FEV_1_/FVC to predicted FEV_1_/FVC value, *V25* maximal expiratory flow at 25% forced VC, *CRP* C-reactive protein, *IgE* immunoglobulin E, *IL* interleukin, *IFN-γ* interferon-gamma, *8-OHdG* 8-hydroxy-2′-deoxyguanosine

**Table 2:** Baseline characteristics of high-concentration and low-concentration toner-exposure groups

Parameters (unit)	High-concentration toner exposure group		Low-concentration toner exposure group		*P*-value
	*n* = 49		*n* = 838		
	M (SD)/%		M (SD)/%		
Age (years)	36.8	(8.3)	38.8	(7.0)	0.100
BMI (kg/m^2^)	22.9	(2.4)	23.4	(3.0)	0.294
Current smoker	53.1%		51.1%		0.883
Prevalence of respiratory diseases (%)					
Asthma	12.2%		9.3%		0.453
Allergic rhinitis	59.2%		50.6%		0.303
Allergic dermatitis	26.5%		20.5%		0.364
Pneumonia	2.7%		0.4%		0.204
Sinusitis	2.0%		2.7%		1.00
Ratio of workers handling harmful substances (%)					
Workers handling dust other than toner	4.1%		2.9%		0.650
Workers handling organic solvent	32.7%		25.9%		0.317
Respiratory function test indicators					
VC (l)	4.3	(0.7)	4.2	(0.6)	0.459
%VC (%)	93.0	(10.3)	92.4	(10.7)	0.695
FEV_1_ (l)	3.5	(0.5)	3.5	(0.5)	0.345
%FEV_1_ (%)	95.1	(10.3)	95.0	(11.2)	0.935
FEV_1_/FVC (%)	83.6	(5.8)	83.0	(5.9)	0.491
%FEV_1_/FVC (%)	100.1	(6.7)	100.1	(6.9)	0.975
V25 (l/s)	1.7	(0.5)	1.6	(0.5)	0.244
%V25 (%)	71.2	(19.7)	68.1	(19.9)	0.481
Biomarkers					
CRP (mg/dl)	0.07	(0.11)	0.09	(0.19)	0.322
IgE (IU/ml)	198.2	(274.4)	201.9	(386.4)	0.953
IL-4 (pg/ml)	19.1	(19.2)	19.2	(29.9)	0.981
IL-6 (pg/ml)	1.15	(0.86)	1.37	(3.25)	0.674
IL-8 (pg/ml)	3.78	(17.85)	2.85	(20.07)	0.773
IFN-γ (IU/ml)	0.08	(0.07)	0.11	(0.13)	< 0.001
8-OHdG/Creatinine (ng/mg)	3.98	(1.64)	3.80	(1.52)	0.738

*M* mean, *SD* standard deviation, *BMI* body mass index, *VC* vital capacity, *%VC* percentage of VC to predicted VC value, *FEV*_*1*_ forced expiratory volume in 1 s, *%FEV*_*1*_ percentage of FEV_1_ to predicted FEV_1_ value, *FEV*_*1*_*/FVC* percentage of forced expiratory volume in 1 s, *%FEV*_*1*_*/FVC* percentage of FEV_1_/FVC to predicted FEV_1_/FVC value, *V25* maximal expiratory flow at 25% forced VC, *CRP* C-reactive protein, *IgE* immunoglobulin E, *IL* interleukin, *IFN-γ* interferon-gamma, *8-OHdG* 8-hydroxy-2′-deoxyguanosine

During the study period, a total of 370 participants (203 toner handlers and 167 non-toner handlers) withdrew from this study. We confirmed the reason for the withdrawal from each participant who withdrew their consent. There was no withdrawal due to the onset of respiratory disease. Table 3 shows the comparison of baseline data between participants who completed the follow-up and those who withdrew from the study. In the toner-handling group, the mean age of participants who withdrew was significantly higher than that of those who completed the follow-up, while the VC, %VC, FEV_1_ and V25 values were significantly lower in the participants who withdrew than in those who completed the follow-up. These significant differences in respiratory function parameters disappeared after adjustment for age. No significant differences were observed in the non-toner-handling group.

**Table 3:** Comparison of baseline characteristics and parameters between participants and those who withdrew from the study

Parameters (unit)	Toner-handling group					Non-toner-handling group				
	Participants		Withdrawal		P-value	Participants		Withdrawal		*P*-value
	*n* = 684		*n* = 203			*n* = 414		*n* = 167		
	M (SD)/%		M (SD)/%			M (SD)/%		M (SD)/%		
Age (years)	38.0	(6.9)	41.2	(7.2)	< 0.001	38.6	(6.1)	38.3	(7.4)	0.629
BMI (kg/m^2^)	23.3	(2.9)	23.5	(3.0)	0.434	23.7	(3.0)	23.6	(2.9)	0.786
Current smoker	52.0%		48.3%		0.379	44.4%		52.7%		0.081
Prevalence of respiratory diseases (%)										
Asthma	9.9%		7.9%		0.416	9.7%		12.0%		0.451
Allergic rhinitis	51.2%		50.7%		0.936	48.6%		47.3%		0.854
Allergic dermatitis	21.6%		18.2%		0.326	22.9%		20.4%		0.581
Pneumonia	0.6%		0.0%		0.579	0.5%		0.0%		1.000
Sinusitis	2.5%		3.4%		0.462	1.4%		3.0%		0.310
Ratio of workers handling harmful substances (%)										
Workers handling dust other than toner	3.8%		0.0%		0.002	0.2%		0.6%		0.493
Workers handling organic solvent	25.6%		28.6%		0.414	2.9%		3.0%		1.000
Respiratory function tests										
VC (l)	4.2	(0.6)	4.1	(0.6)	< 0.001	4.2	(0.6)	4.2	(0.6)	0.606
%VC (%)	92.9	(107.0)	90.7	(10.5)	0.012	91.8	(9.8)	91.9	(9.9)	0.895
FEV_1_ (l)	3.5	(0.5)	3.4	(0.5)	< 0.001	3.5	(0.5)	3.5	(0.5)	0.759
%FEV_1_ (%)	95.3	(11.3)	93.9	(10.7)	0.120	94.6	(10.8)	94.2	(10.9)	0.700
FEV_1_/FVC (%)	83.1	(6.1)	82.8	(5.5)	0.552	83.0	(6.0)	82.7	(6.0)	0.596
%FEV_1_/FVC (%)	100.0	(6.9)	100.5	(6.9)	0.344	100.3	(7.2)	99.7	(7.0)	0.410
V25 (l/s)	1.6	(0.5)	1.5	(0.4)	0.002	1.6	(0.5)	1.6	(0.6)	0.669
%V25 (%)	68.5	(20.2)	67.4	(18.9)	0.500	67.3	(18.3)	67.4	(19.9)	0.974
Biomarkers										
CRP (mg/dl)	0.08	(0.16)	0.11	(0.24)	0.135	0.10	(0.19)	0.10	(0.24)	0.935
IgE (IU/ml)	194.1	(386.2)	229.0	(360.2)	0.313	206.4	(436.5)	252.7	(429.4)	0.251
IL-4 (pg/ml)	19.9	(31.7)	16.6	(18.3)	0.219	10.8	(35.8)	10.7	(14.4)	0.988
IL-6 (pg/ml)	1.3	(3.2)	1.4	(3.0)	0.763	1.3	(5.5)	0.9	(0.8)	0.430
IL-8 (pg/ml)	3.2	(22.0)	1.8	(9.2)	0.449	1.1	(0.7)	1.2	(1.0)	0.365
IFN-γ (IU/ml)	0.1	(0.1)	0.1	(0.1)	0.902	0.1	(0.1)	0.1	(0.1)	0.220
8- OHdG/Creatinine (ng/mg)	3.9	(1.4)	3.9	(1.9)	0.883	3.8	(1.5)	3.8	(1.4)	0.692

*M* mean, *SD* standard deviation, *BMI* body mass index, *VC* vital capacity, *%VC* percentage of VC to predicted VC value, *FEV*_*1*_ forced expiratory volume in 1 s, *%FEV*_*1*_ percentage of FEV_1_ to predicted FEV_1_ value, *FEV*_*1*_*/FVC* percentage of forced expiratory volume in 1 s, *%FEV*_*1*_*/FVC* percentage of FEV_1_/FVC to predicted FEV_1_/FVC value, *V25* maximal expiratory flow at 25% forced VC, *CRP* C-reactive protein, *IgE* immunoglobulin E, *IL* interleukin, *IFN-γ* interferon-gamma, *8-OHdG* 8-hydroxy-2′-deoxyguanosine

### Chest X-ray examination

In the baseline survey, none of the participants had lung fibrosis that was 1/1 or greater on a 12-point profusion scale using chest X-ray. A total of 11,563 chest X-ray examinations were conducted in the 10-year follow-up period (7368 chest X-ray photographs in the toner-handling group included 461 photographs of high-concentration toner exposure, 6925 photographs of low-concentration toner exposure, and 4177 chest X-ray photographs in the non-toner-handling group). One participant in the low-concentration toner exposure group scored 1/1 on the 12-point scale in the second follow-up survey, while one participant in the non-toner-handling group scored 1/2 in the seventh follow-up survey. However, these findings disappeared in the subsequent follow-up surveys.

### Respiratory function and serum and urinary biomarkers

In the baseline survey, the data of 186 participants for serum and urinary biomarkers (toner-handling group, 169; non-toner-handling group, 17) could be unreliable due to inappropriate blood or urine sample collection procedures or damage of the samples during transportation. Therefore, these data were excluded from this study analysis.

We discontinued the measurements of four cytokines (IL-4, IL-6, IL-8, and IFN-γ) by 2008 (fifth follow-up) and excluded them from the longitudinal analysis. For IL-4 and IL-8, no significant differences were found between the toner-handling group and non-toner- handling group in any of the years up to the fifth year of the study. For IL-6 and IFN-γ, there were some years in which significant differences were observed between the two groups, but these differences were not consistent and did not exceed the reference value; they were therefore considered to be of low clinical significance. Particularly for IL-8 and IFN-γ, 7664 measurements of IL-8 and IFN-γ conducted from the baseline survey until the fifth follow-up survey; 6746 measurements of IL-8 (88%); and 7128 measurements of IFN-γ (93%), were below the LOD. Based on these results, we considered that the four cytokines did not reflect the biological effects of the toner exposure. The means values for each year, of these four cytokines in both groups are provided in an additional table file [see Additional file 1].

### Panel data analysis

**Fig. 1:**
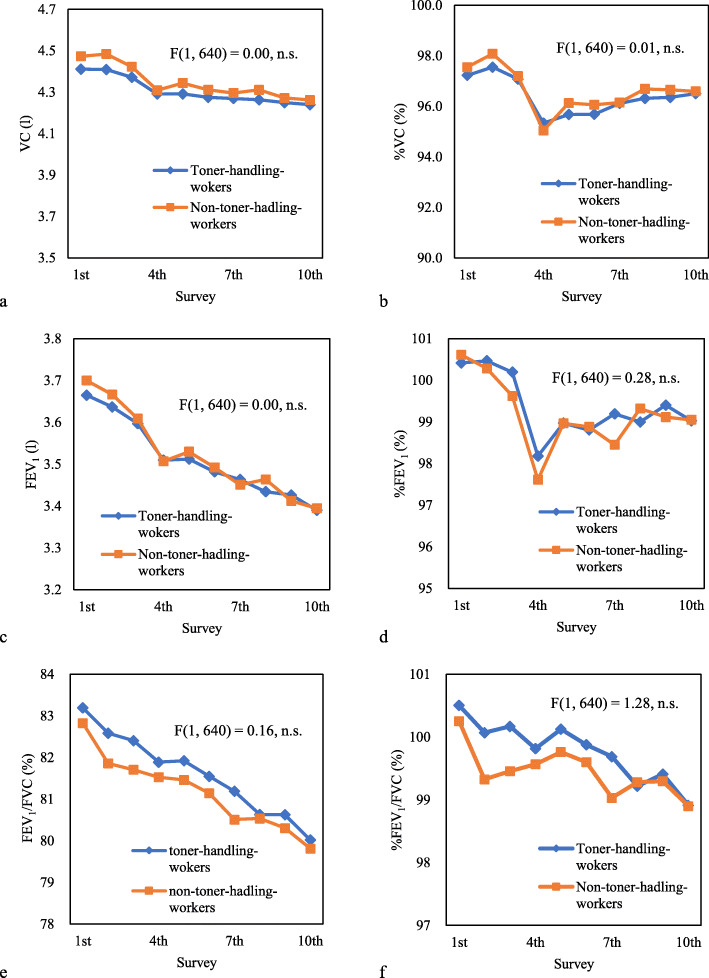
The mean value profiles of the respiratory function test. Mean value of each parameter of the respiratory function test for each study year and the F value of the between-subjects effect of toner-handling work obtained by the two-way repeated measurement analysis of variance; **a** mean value profiles of VC, **b** mean value profiles of %VC, **c** mean value profiles of FEV_1_, **d** mean value profiles of %FEV_1_, **e** mean value profiles of FEV_1_/FVC, **f** mean value profiles of %FEV_1_/FVC. VC: vital capacity, %VC: percentage of VC to predicted VC value, FEV_1_: forced expiratory volume in 1 s, %FEV_1_: percentage of FEV_1_ to predicted FEV_1_ value, FEV_1_/FVC: percentage of forced expiratory volume in 1 s, %FEV_1_/FVC: percentage of FEV_1_/FVC to predicted FEV_1_/FVC value

**Fig. 2:**
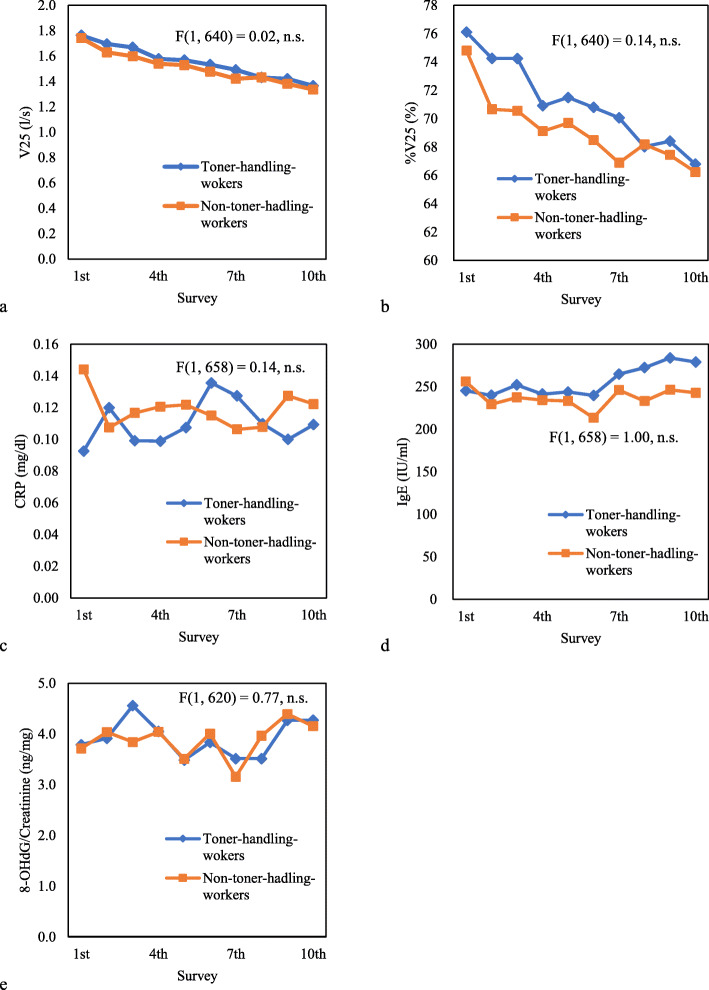
The mean value profiles of V25, %V25 and biomarkers. The figure shows the profile of the mean values of V25, %V25, CRP, Ig E, and creatinine-corrected 8-OHdG for each study year, and F-value of the between-subjects effects of toner-handling work obtained by the two-way repeated measurement analysis of variance. **a** The mean value profiles of V25, **b** mean value profiles of %V25, **c** mean value profiles of CRP, **d** mean value profiles of Ig E, and **e** mean value profiles of creatinine-corrected 8-OHdG. V25: maximal expiratory flow at 25% FVC, %V25: percentage of V25 to predicted V25 value, CRP: C-reactive protein, IgE: immunoglobulin E, 8-OHdG: 8-hydroxy-2′-deoxyguanosine

Fig. 1 shows the mean value profiles of VC, %VC, FEV_1_, %FEV_1_, FEV_1_/FVC, and %FEV_1_/FVC during the study period in the toner-handling and non-toner-handing groups. Figure 2 shows the profile of the mean, and F-values of the remaining parameters. A two-way repeated measures analysis comparing the two groups showed no significant between-subjects effect of toner-handling work for all parameters.

### Longitudinal data analysis

The health effects attributed to the toner exposure were indicated as the differences in the yearly changes in parameters between the toner-handling group (subgroups included) and non-toner handling group. The differences in the yearly changes were calculated as estimated values of the coefficient of interaction between toner-handling work and survey year in LMM. Table 4 shows the estimated health effects of toner exposure using models 1 and 2, and also shows the AIC of models 1 and 2. Model 2 fitted better than model 1 in all parameters. Therefore, model 3 was analyzed using the random intercept and random slope models. For the analysis between the three groups (high-concentration toner exposure group, low-concentration toner exposure group, and non-toner-handling group), numerical calculations of VC, %VC, and FEV_1_ did not converge in the random-intercept model. For all the parameters, the random intercept and slope model showed a better fit than the random intercept model. Hence, we also adopted the random intercept and slope model for model 4.

**Table 4:** Analysis of the health effects of toner exposure without adjustment for confounders (models 1 and 2)

Parameters (unit)	Difference in yearly change in each parameter between the toner-handling group and non-toner-handling group (model 1)					Difference in yearly change in each parameter between the toner-handling group and non-toner-handling group (model 2)				
	Estimate	95% CI		*P*-value	AIC	Estimate	95% CI		*P*-value	AIC
Respiratory function tests										
VC (l)	0.0009	−0.002	0.003	0.459	− 3786.6	− 0.003	− 0.005	0.0002	0.067	− 4375.4
%VC (%)	0.004	− 0.05	0.06	0.892	74,963.3	−0.07	− 0.1	− 0.004	0.037	74,441.0
FEV_1_ (l)	0.0001	−0.002	0.002	0.925	− 7999.7	−0.001	− 0.004	0.001	0.272	− 8462.7
%FEV_1_ (%)	0.02	− 0.05	0.08	0.608	76,776.5	−0.02	− 0.09	0.05	0.568	76,306.5
FEV_1_/FVC (%)	−0.003	− 0.04	0.03	0.866	60,893.8	−0.002	− 0.04	0.04	0.903	60,721.0
%FEV_1_/FVC (%)	0.006	−0.04	0.05	0.808	65,957.3	0.008	−0.04	0.06	0.727	65,790.5
V25 (l/s)	0.0005	−0.003	0.004	0.747	4573.4	0.0002	−0.004	0.004	0.921	4303.0
%V25 (%)	−0.06	− 0.2	0.10	0.457	102,127.6	−0.02	− 0.20	0.15	0.784	101,834.2
Biomarkers										
CRP (mg/dl)	0.0007	−0.002	0.003	0.635	3626.3	0.0006	−0.002	0.003	0.658	3620.2
IgE (IU/ml)	3.2	−1.6	7.9	0.190	177,623.3	4.6	−1.00	10.1	0.108	175,403.0
8-OHdG/Creatinine (ng/mg)	−0.01	− 0.03	0.0009	0.065	45,402.9	−0.01	−0.03	0.003	0.115	45,352.5

Dependent variables comprised the parameters of respiratory function tests and the values of biomarkers in both models 1 and 2. In model 1, we treated toner-handling work, the survey year, and the interaction between toner-handling work and survey year as fixed effects and treated only the individual differences at baseline as the random effect (random intercept model). In model 2, we treated certain individual differences during the observation period and the individual differences at baseline as random effects (random intercept and slope models)

*CI* confidence interval, *AIC* Akaike’s Information Criterion, *VC* vital capacity, *%VC* percentage of VC to predicted VC value, *FEV*_*1*_ forced expiratory volume in 1 s, *%FEV*_*1*_ percentage of FEV_1_ to predicted FEV_1_ value, *FEV*_*1*_*/FVC* percentage of forced expiratory volume in 1 s, *%FEV*_*1*_*/FVC* percentage of FEV_1_/FVC to predicted FEV_1_/FVC value, *V25* maximal expiratory flow at 25% forced VC, *CRP* C-reactive protein, *IgE* immunoglobulin E, *8-OHdG* 8-hydroxy-2′-deoxyguanosine

Table 5 shows the estimated health effects of toner exposure using model 3. Table 6 shows the differences in the yearly changes in parameters among the high-concentration toner exposure group, low-concentration toner exposure group, and non-toner-handling group using model 4. The yearly changes in each parameter in the non-toner handling group, corresponding to the estimated coefficients for the study year in the LMM, are also shown in Tables 5 and 6. We observed significant effects only in %VC.

**Table 5:** Analysis of the health effects of toner exposure with adjustment for confounders (model 3)

Parameters (unit)	Difference in yearly change in each parameter between the toner-handling group and non-toner-handling group				Yearly change in each parameter of the non-toner-handling group				
	Estimate	95% CI		*P*-value	Estimate	95% CI		*P*-value	AIC
Respiratory function tests									
VC (l)	−0.001	− 0.004	0.001	0.294	−0.01	− 0.01	− 0.01	< 0.001	− 5289.3
%VC (%)	− 0.04	− 0.1	0.02	0.188	0.2	0.1	0.2	< 0.001	73,416.2
FEV_1_ (l)	0.00003	−0.002	0.002	0.982	−0.02	−0.02	− 0.02	< 0.001	− 9986.4
%FEV_1_ (%)	0.02	−0.05	0.08	0.616	0.1	0.09	0.2	< 0.001	74,397.2
FEV_1_/FVC (%)	0.004	−0.03	0.04	0.843	−0.3	−0.3	− 0.3	< 0.001	60,385.0
%FEV_1_/FVC (%)	0.02	−0.03	0.06	0.510	−0.09	−0.1	− 0.05	< 0.001	65,542.7
V25 (l/s)	0.001	−0.003	0.005	0.607	−0.03	−0.03	− 0.03	< 0.001	3467.3
%V25 (%)	0.02	−0.1	0.2	0.781	−0.4	−0.6	− 0.3	< 0.001	101,019.6
Biomarkers									
CRP (mg/dl)	0.0004	−0.002	0.003	0.809	0.0004	−0.002	0.003	0.720	3574.3
IgE (IU/ml)	4.8	−1.0	10.6	0.107	−1.6	−6.1	3.0	0.500	174,528.7
8-OHdG/Creatinine (ng/mg)	−0.009	−0.03	0.006	0.235	0.03	0.02	0.05	< 0.001	44,980.9

In model 3, dependent variables consisted of the parameters of respiratory function tests and the values of biomarkers. We used the higher goodness-of-fit model in models 1 and 2 and adjusted the model using age at baseline, body mass index, smoking, asthma, allergic rhinitis, pneumonia, sinusitis, non-toner-handling work, and organic solvent-handling work as confounding factors

*CI* confidence interval, *AIC* Akaike’s Information Criterion, *VC* vital capacity, *%VC* percentage of VC to predicted VC value, *FEV*_*1*_ forced expiratory volume in 1 s, *%FEV*_*1*_ percentage of FEV_1_ to predicted FEV_1_ value, *FEV*_*1*_*/FVC* percentage of forced expiratory volume in 1 s, *%FEV*_*1*_*/FVC* percentage of FEV_1_/FVC to predicted FEV_1_/FVC value, *V25* maximal expiratory flow at 25% forced VC, *CRP* C-reactive protein, *IgE* immunoglobulin E, *IL* interleukin, *IFN-γ* interferon-gamma, *8-OHdG* 8-hydroxy-2′-deoxyguanosine

**Table 6:** Analysis of the health effects of toner exposure with adjustment for confounders (model 4)

Parameters (unit)	Difference in yearly change in each parameter between the low-concentration toner exposure group and non-toner-handling group				Difference in yearly change in each parameter between the high-concentration toner exposure group and non-toner-handling group				Yearly change in each parameter of the non-toner-handling group				
	Estimate	95% CI		*P*-value	Estimate	95% CI		*P*-value	Estimate	95% CI		*P*-value	AIC
Respiratory function tests													
VC (l)	−0.001	−0.004	0.002	0.378	−0.005	−0.01	0.002	0.159	−0.007	−0.01	− 0.005	< 0.001	− 5278.0
%VC (%)	− 0.03	−0.10	0.03	0.284	−0.2	−0.3	− 0.003	0.045	0.2	0.1	0.2	< 0.001	73,413.9
FEV_1_ (l)	0.0001	−0.002	0.002	0.954	−0.001	−0.007	0.005	0.824	−0.02	−0.02	− 0.02	< 0.001	− 9973.4
%FEV_1_ (%)	0.02	−0.05	0.08	0.614	0.01	−0.2	0.2	0.898	0.1	0.09	0.2	< 0.001	74,937.0
FEV_1_/FVC (%)	0.001	−0.04	0.04	0.964	0.05	−0.05	0.1	0.325	−0.3	−0.3	− 0.3	< 0.001	60,386.6
%FEV_1_/FVC (%)	0.01	−0.04	0.06	0.670	0.10	−0.02	0.23	0.108	−0.09	−0.1	− 0.05	< 0.001	65,542.2
V25 (l/s)	0.001	−0.003	0.005	0.640	0.002	−0.007	0.01	0.448	−0.03	−0.03	− 0.03	< 0.001	3479.7
%V25 (%)	0.02	−0.2	0.2	0.861	0.2	−0.3	0.6	0.470	−0.4	−0.6	− 0.3	< 0.001	10,1016.2
Biomarkers													
CRP (mg/dl)	0.0002	−0.003	0.003	0.876	0.002	−0.005	0.010	0.558	0.0004	−0.2	−0.08	0.717	3589.0
Ig E (IU/ml)	4.9	−1.0	10.7	0.106	3.5	−12.4	19.4	0.206	−1.6	−6.1	3.0	0.500	17,4511.9
8-OHdG/Creatinine (ng/mg)	−0.01	−0.03	0.006	0.898	0.003	−0.04	0.04	0.758	0.033	0.02	0.05	< 0.001	44,987.9

In model 4, dependent variables consisted of the parameters of respiratory function tests and the values of biomarkers. We compared the high-concentration toner exposure group, low-concentration toner exposure group, and non-toner-handling group and adjusted the model using age at baseline, body mass index, smoking, asthma, allergic rhinitis, pneumonia, sinusitis, non-toner-handling work, and organic solvent-handling work as confounding factors

*CI* confidence interval, *AIC* Akaike’s Information Criterion, *VC* vital capacity, *%VC* percentage of VC to predicted VC value, *FEV*_*1*_ forced expiratory volume in 1 s, *%FEV*_*1*_ percentage of FEV_1_ to predicted FEV_1_ value, *FEV*_*1*_*/FVC* percentage of forced expiratory volume in 1 s, *%FEV*_*1*_*/FVC* percentage of FEV_1_/FVC to predicted FEV_1_/FVC value, *V25* maximal expiratory flow at 25% forced VC, *CRP* C-reactive protein, *IgE* immunoglobulin E, *8-OHdG* 8-hydroxy-2′-deoxyguanosine

As for %VC, the analysis in model 3 comparing the whole toner-handling group with the non-toner-handling group showed no significant difference in yearly changes. In model 4, analyzed using the three levels of toner exposure, the difference in yearly changes between the low-concentration toner exposure group and non-toner-handling group was not significant, while a significant difference was observed between the high-concentration toner exposure group and non-toner-handling group. %VC showed a significant upward trend in the non-toner-handling group. When the analysis using the LMM adjusted for the same confounding factors as those in models 3 and 4 was performed respectively for each exposure concentration group, the yearly change in each group was as follows: high-concentration toner exposure group, − 0.11% (95% confidence interval [CI], − 0.29 to 0.08; *P* = 0.250); low-concentration toner exposure group, 0.13% (95% CI, 0.09–0.17; *P* < 0.001); and non-toner-handling group, 0.15% (95% CI, 0.01–0.20; P < 0.001).

## Discussion

To clarify the health effects of toner exposure, we explored the differences in yearly changes in the parameters of chest X-ray examinations, respiratory function indicators measured by spirometry and flow-volume curve, and biomarkers of inflammation, allergy, and oxidative stress, between tone-handling workers and non-toner-handling workers. We did not observe any increased rate of onset of lung fibrosis associated with toner-handling work in the chest X-ray examinations. Furthermore, almost all the yearly changes in respiratory function indicators and serum and urinary biomarkers were similar between the toner-handling group and non-toner-handling group. On the other hand, the yearly changes in %VC differed depending on the presence or absence of toner-handling work.

Some cross-sectional studies have evaluated the health effects of toner-printing work at copy centers. In a survey conducted at a copy center in India, a significant increase in serum IL-8 was observed in toner-printing workers compared with non-toner-printing workers [32]. Another survey in the United States reported a transient increase in urinary 8-OHdG levels in healthy participants who spent time in copy centers for several days [33]. Moreover, a cross-sectional study of Iranian copy centers reported that the FVC and FEV_1_ were significantly lower in toner-printing workers than in non-toner-printing groups [34]. These reports suggest that toner-printing work at copy centers may cause inflammatory reactions, oxidative stress, and deterioration of respiratory function. In general, exposure to toner particles may occur in workers in copy centers only when the toner is not fused to the paper owing to printing failure or when toner particles leak during toner cartridge replacement. However, these exposures, secondary to printing failure and copy center work, likely occur at a low rate. The participants in the studies mentioned above might have very little direct exposure to toner particles. These copy center studies were designed primarily to investigate the health effects of PM emitted during printing. Therefore, in these studies, it would have been difficult to evaluate only the health effects of toner particle exposure excluding the effects of exposure to other PMs related to the printing process. Furthermore, since cross-sectional studies are influenced by factors other than the exposure, such as individual differences, the causal relationship between work at the copy center and changes in the levels of respiratory function and biomarkers cannot be clearly defined.

Several epidemiological cohort studies aimed at investigating the health effects of toner particle exposure have been conducted at toner manufacturing plants [12–15]. No significant differences were reported between the toner-handling and non-toner-handling groups in terms of the development of new-onset lung fibrosis on follow-up chest X-ray examination. Regarding respiratory function, yearly changes and the occurrence of outliers of peak expiratory flow rate (PEFR), VC, %VC, FEV_1_, %FEV_1_, FVC, and FEV_1_/FVC have been investigated in these studies, but no clear differences have been reported between the toner-handling group and the non-toner-handling group. Regarding biomarkers, the relationship between serum CRP, serum IgE, urinary 8-OHdG, etc., and toner particle exposure have also been investigated. However, these studies reported that the number of occurrence of outliers and the yearly changes in the values in the toner-handling group had a close resemblance to those in the non-toner-handling group. Remarkable differences in changes in respiratory function among individuals over time have been reported [35–37]. Furthermore, lifestyle habits such as smoking and alcohol consumption have been reported to affect urinary 8-OHdG levels [38–40]. Therefore, the parameters evaluated in this study could have been influenced not only by toner exposure but also by individual differences. However, the previous cohort studies were limited in terms of the analysis that considered inter-individual differences. The strength of this study is that we performed analyses that modeled inter-individual differences as a random effect.

The chest computed tomography (CT) is considered to be superior to the chest X-ray in detecting early pulmonary changes of respiratory disease [41–43]. On the other hand, in Japan, chest X-ray is originally included in the annual medical examinations that employers are required to conduct for workers under the law, and its information can be obtained without additional radiation exposure to the workers. For these reasons, we chose the chest X-rays as the images to inspect in observing the development of respiratory disease due to the effect of toner exposure. Although it would have been difficult to perform the chest CT on all the participants in this study, more detailed information might have been obtained if a chest CT had been performed on those with abnormalities on the chest X-ray.

In this study, we hypothesized that each parameter of the respiratory functions in the toner-handling group decreased more than the usual decrease in respiratory functions with aging if there were chronic health effects owing to toner exposure. We also hypothesized that the higher the toner exposure concentration, the greater the decline over time, in each parameter, according to the dose-response relationship. However, this study showed that the %VC in the non-toner-handling group and low-concentration toner exposure group increased over time, while those in the high-concentration toner exposure group did not change significantly.

%VC showed different changes over time depending on the toner exposure status. %VC increased significantly over time in the low-concentration toner exposure group and the non-toner handling group, while no change over time was observed in the high-concentration toner exposure group. Meanwhile, the significant decrease in VC was observed for all the groups regardless of the degree of toner handling. The increase in the %VC appears to be interest; however, it was thought to be due to the predicted VC. In this cohort, VC was estimated to decrease by 0.007 L per year regardless of the toner exposure condition as shown in Table 6. On the other hand, the predicted VC obtained from the prediction equation [21] decreased to a greater extent. For example, the predicted VC of a 170 cm, 40-year old man is estimated to decrease by 0.16 L over 10 years, but was only 0.07 L in this cohort. %VC did indeed increase in the low-concentration toner exposure and non-handling groups, but the increases were only 0.13 and 0.15% per year, respectively. The non-significant statistical change in %VC in the high-concentration toner exposure group may be owing to the small amount of change and the lack of statistical power due to the smaller sample size in the high-concentration toner exposure group compared to the other two groups.

In terms of the low toxicity associated with toner exposure, the findings of the chest X-ray examinations and the yearly changes in respiratory function indicators and serum and urinary biomarkers in this study are consistent with the trend indicated by the findings of inhalation exposure studies in animals [44–46].

### Limitation and future direction

This study has several limitations. First, there was a problem with the sample size. We attempted to determine the dose-response relationship between exposure levels and health effects by comparing various parameters among the non-toner-handling group, low-concentration toner exposure group, and high-concentration toner exposure group. However, the sample size for the high-concentration toner exposure group was 49, which may have been too small. While this study had a reasonably sufficient power to detect distinct outcome discrepancy between the toner-handling group and non-toner-handling group, this may not have been the case for the three-group comparison. According to Cohen, assuming an 80% power, a small effect size, and 5% levels of significance, a total of 969 cases (323 cases per group) are needed for a three-group mean comparison [47]. Recently, several other epidemiological studies on the health effects of toner exposure have been conducted [48, 49]. A pooled analysis using the data from these studies may be helpful in elucidating the dose-response relationship. Second, we may need to continue following-up these toner-handling workers, since our observation period may have been short. In particular, it takes a long time for lung fibrosis or pulmonary obstructive disorder to develop. Third, the health effects of toner exposure could have been underestimated due to the healthy worker bias [50]. As toner-handling work may involve higher physical load than non-toner-handling work, healthy workers might have been preferentially assigned to toner-handling work. Fourth, the actual level of toner exposure may have been lower than expected. At the study site, adequate ventilation was in operation to control dust scattering, and workers that were engaged in frequent toner-handling works wore appropriate respiratory masks. Although there could have been an overestimation of the toner exposure, personal exposure measurements had to be performed outside the mask to protect the health of the toner-handling workers during measurement.

## Conclusions

After 10 years of observation of participants under 50 years of age who were occupationally exposed to toner particles, we found no obvious health effect of toner handling work on the onset of lung fibrosis, deterioration of respiratory function, or increases in the values of biomarkers for inflammation, allergies, and oxidative stress. Our study shows that toner-handling work has minimal adverse effects on the respiratory system in work environments where dust aerosolization is sufficiently controlled by ventilation.

## Supplementary Information

**Additional file 1.** Comparison of discontinued biomarkers between the toner-handling group and non-toner-handling groups.

## Data Availability

The datasets used and analyzed during the current study are available from the corresponding author on reasonable request.

## Abbreviations

AIC: Akaike’s Information Criterion
CI: confidence interval
CRP: C-reactive protein
CT: Computed tomography
FEV_1_: Forced expiratory volume in 1 s
%FEV_1_: Percentage of FEV_1_ to predicted FEV1 value
FEV_1_/FVC: Percentage of forced expiratory volume in 1 s to forced vital capacity
%FEV_1_/FVC: Percentage of FEV_1_/FVC to predicted FEV_1_/FVC value
FVC: Forced vital capacity
IgE: Immunoglobulin E
IFN-γ: Interferon-gamma
IL: Interleukin
LMM: Linear mixed model
LOD: Limit of detection
8-OHdG: 8-hydroxy-2′-deoxyguanosine
PEFR: Peak expiratory flow rate
PM: Particulate matter
TLV-TWA: Threshold limit value–time-weighted average
TWA-8 h: Mean 8-h time-weighted average at baseline survey
V25: Maximal expiratory flow at 25% FVC
%V25: Percentage of V25 to predicted V25 value
VC: Vital capacity
%VC: Percentage of VC to predicted VC value

## References

[1] Gallardo M, Romero P, Sánchez-Quevedo MC, López-Caballero JJ (1994). Siderosilicosis due to photocopier toner dust. Lancet..

[2] Armbruster C, Dekan G, Hovorka A (1996). Granulomatous pneumonitis and mediastinal lymphadenopathy due to photocopier toner dust. Lancet..

[3] Wieriks J (1996). Photocopier toner dust and lung disease. Lancet..

[4] Wittczak T, Walusiak J, Ruta U, Pałczynski C (2003). Occupational asthma and allergic rhinitis due to xerographic toner. Allergy..

[5] He C, Morawska L, Taplin L (2007). Particle emission characteristics of office printers. Environ Sci Technol.

[6] Kagi N, Fujii S, Horiba Y, Namiki N, Ohtani Y, Emi H (2007). Indoor air quality for chemical and ultrafine particle contaminants from printers. Build Environ.

[7] Namiki N, Otani Y, Fujii S, Kagi N (2006). Characterization of emission of ultrafine particles from office printers [in Japanese]. Earozoru Kenkyu.

[8] Barthel M (2011). XRF-analysis of fine and ultrafine particles emitted from laser printing devices. Environ Sci Technol..

[9] Schripp T, Wensing M, Salthammer T, Uhde E (2009). Emission of VOCs and SVOCs from electronic devices and office equipment. Organic indoor air pollutants.

[10] Sung G, Ha S, Kwon SB, Kim T (2017). Reduction of ultrafine particles emission from office laser printers. J Aerosol Sci.

[11] Wang ZM, Wagner J, Wall S (2011). Characterization of laser printer nanoparticle and VOC emissions, formation mechanisms, and strategies to reduce airborne exposures. Aerosol Sci Technol.

[12] Nakadate T, Yamano Y, Adachi C, Kikuchi Y, Nishiwaki Y, Nohara M (2006). A cross sectional study of the respiratory health of workers handling printing toner dust. Occup Environ Med.

[13] Yanagi N, Kitamura H, Mizuno M, Hata K, Uchiyama T, Kuga H (2014). A 4-year follow-up cohort study of the respiratory function tests in toner-handling workers. Saf Health Work.

[14] Ikegami K, Hasegawa M, Ando H, Hata K, Kitamura H, Ogami A, Higashi T. A cohort study of the acute and chronic respiratory effects of toner exposure among handlers: a longitudinal analysis from 2004 to 2013. Ind Health 2016;54(5):448–459. doi: 10.2486/indhealth.2015-0202. PubMed: 27021062. 10.2486/indhealth.2015-0202 PMC5054286 27021062

[15] Nakadate T, Yamano Y, Yamauchi T, Okubo S, Nagashima D (2018). Assessing the chronic respiratory health risk associated with inhalation exposure to powdered toner for printing in actual working conditions: a cohort study on occupationally exposed workers over 10 years. BMJ Open.

[16] Ternunuma N, Ikegami K, Kitamura H, Ando H, Kurosaki S, Masuda M, Kochi T, Yanagi N, Ogami A, Higashi T (2019). A cohort study on respiratory symptoms and diseases caused by toner-handling work: longitudinal analyses from 2003 to 2013. Atmosphere..

[17] Ministry of Labour, Japan. Jinpai Hyoujyun X-ray films (Standard X-ray films for diagnosis of pneumoconiosis) [in Japanese]. Tokyo, Japan: Japan Safety and Health Association; 1982.

[18] Department of Health and Safety, Ministry of Labour, Japan. Jinpai Shinsa handbook (Pneumoconiosis examination handbook) [in Japanese]. Tokyo, Japan: Japan Safety and Health Association; 1978.

[19] The International Labour Organization (ILO). Guidelines for use of the pneumoconiosis. revised 2000 ed. Geneva, Switzerland: International Labor Office; 2002.

[20] Miller MR, Hankinson J, Brusasco V, Burgos F, Casaburi R, Coates A (1995). Standardization of spirometry, 1994 update. American Thoracic Society. Am J Respir Crit Care Med.

[21] Kubota M, Kobayashi H, Quanjer PH, Omori H, Tatsumi K, Kanazawa M (2014). Reference values for spirometry, including vital capacity, in Japanese adults calculated with the LMS method and compared with previous values. Respir Investig.

[22] Japanese Respiratory Society (2001). Reference values of spirogram and arterial blood gas analysis in Japanese [in Japanese]. Ann Jpn Respir Soc.

[23] Matsuda Y, Harada Y, Tanno Y (2013). State of toner exposure of workers who handle toners. J Occup Health.

[24] Matsumura Y, Suzuki C, Ishiyama T (2002). Development of EA-toner (emulsion aggregation toner) for high quality and oil-less printing [in Japanese]. Fuji Xerox Tech Rep.

[25] Terunuma N, Kurosaki S, Kitamura H, Hata K, Ide R, Kuga H (2009). Cross-sectional study on respiratory effect of toner exposure. Hum Exp Toxicol..

[26] Kitamura H, Terunuma N, Kurosaki S, Hata K, Masuda M, Kochi T (2014). A cohort study on self-reported respiratory symptoms of toner-handling workers: cross-sectional and longitudinal analysis from 2003 to 2008. Biomed Res Int.

[27] Kitamura H, Terunuma N, Kurosaki S, Hata K, Masuda M, Kochi T (2015). A cohort study of toner-handling workers on inflammatory, allergic, and oxidative stress markers: cross-sectional and longitudinal analyses from 2003 to 2008. Hum Exp Toxicol..

[28] Kitamura H, Terunuma N, Kurosaki S, Hata K, Masuda M, Kochi T (2015). A cohort study using pulmonary function tests and X-ray examination in toner-handling workers: cross-sectional and longitudinal analyses from 2003 to 2008. Hum Exp Toxicol..

[29] The American Conference of Governmental Industrial Hygienists (ACGIH). Documentation of the Threshold Limit Values and Biological Exposure Indices, 7th ed.; ACGIH: Cincinnati, Ohio, USA; 2001.

[30] Fitzmurice GM, Laird NM, Ware JH. Applied longitudinal analysis. 2nd ed: Wiley; 2011.

[31] Kitamura H, Terunuma N, Kurosaki S, Hata K, Ide R, Kuga H (2009). Cross-sectional study on respiratory effect of toner-exposed work in manufacturing plants, Japan: pulmonary function, blood cells, and biochemical markers. Hum Exp Toxicol.

[32] Elango N, Kasi V, Vembhu B, Poornima JG (2013). Chronic exposure to emissions from photocopiers in copy shops causes oxidative stress and systematic inflammation among photocopier operators in India. Environ Health.

[33] Khatri M, Bello D, Gaines P, Martin J, Pal AK, Gore R (2013). Nanoparticles from photocopiers induce oxidative stress and upper respiratory tract inflammation in healthy volunteers. Nanotoxicology..

[34] Karimi A, Eslamizad S, Mostafaee M, Momeni Z, Ziafati F, Mohammadi S (2016). Restrictive pattern of pulmonary symptoms among photocopy and printing workers: a retrospective cohort study. J Res Health Sci.

[35] Nakadate T, Kagawa J (1992). Between- and within-subject variability of pulmonary function. Four year follow-up of adult males [in Japanese]. Sangyo Igaku.

[36] Nishitsuji M, Fujimura M, Oribe Y, Kimura H, Nomura S, Yoshimoto A (2003). Influence of smoking on longitudinal decline in one-second forced expiratory volume in clinically healthy Japanese men: a longitudinal study. Nihon Kokyuki Gakkai Zasshi.

[37] Agusti A, Calverley PM, Celli B, Coxson HO, Edwards LD, Lomas DA (2010). Characterisation of COPD heterogeneity in the eclipse cohort. Respir Res.

[38] Kasai H, Iwamoto-Tanaka N, Miyamoto T, Kawanami K, Kawanami S, Kido R (2001). Lifestyle and urinary 8-hydroxydeoxyguanosine, a marker of oxidative DNA damage: effects of exercise, working conditions, meat intake, body mass index, and smoking. Jpn J Cancer Res.

[39] Wu LL, Chiou CC, Chang PY, Wu JT (2004). Urinary 8-OHdG: a marker of oxidative stress to DNA and a risk factor for cancer, atherosclerosis and diabetics. Clin Chim Acta.

[40] Irie M, Tamae K, Iwamoto-Tanaka N, Kasai H (2005). Occupational and lifestyle factors and urinary 8-hydroxydeoxyguanosine. Cancer Sci.

[41] Sone S (2000). Characteristics of small lung cancers invisible on conventional chest radiography and detected by population based screening using spiral CT. Br J Radio.

[42] Soda H (1993). Limitation of annual screening chest radiography for the diagnosis of lung cancer. A retrospective study. Cancer Sci.

[43] Altorki N, Kent M, Pasmantier M (2001). Detection of early-stage lung cancer: computed tomographic scan or chest radiograph?. J Thorac Cardiovasc Surg.

[44] Morimoto Y, Hirohashi M, Kasai T, Oyabu T, Ogami A, Myojo T (2009). Effect of polymerized toner on rat lung in chronic inhalation study. Inhal Toxicol.

[45] Morimoto Y, Kim H, Oyabu T, Hirohashi M, Nagatomo H, Ogami A (2005). Negative effect of long-term inhalation of toner on formation of 8-hydroxydeoxyguanosine in DNA in the lungs of rats in vivo. Inhal Toxicol.

[46] Muhle H, Bellmann B, Creutzenberg O, Koch W, Dasenbrock C, Ernst H (1998). Pulmonary response to toner, TiO2 and crystalline silica upon chronic inhalation exposure in Syrian golden hamsters. Inhal Toxicol.

[47] Cohen J (1992). A power primer. Psychol Bull.

[48] Hasegawa M, Kitamura H, Ikegami K, Masuda M, Kakiuchi N, Matsushita T (2018). The respiratory effects of toner exposure according to long-term occupational toner handling history: A longitudinal analysis, 2004–2013. Int J Occup Med Environ Health.

[49] Kitamura H, Ogami A, Myojo T, Oyabu T, Ikegami K, Hasegawa M (2019). Health effects of toner exposure among Japanese toner-handling workers: a 10-year prospective cohort study. J UOEH.

[50] Delgado-Rodríguez M, Llorca J (2004). Bias. J Epidemiol Commun Health.

